# *Neotrombicula inopinata* (Acari: Trombiculidae) – a possible causative agent of trombiculiasis in Europe

**DOI:** 10.1186/1756-3305-7-90

**Published:** 2014-03-03

**Authors:** Alexandr A Stekolnikov, Paula Santibáñez, Ana M Palomar, José A Oteo

**Affiliations:** 1 Zoological Institute, Russian Academy of Sciences, Universitetskaya Embankment 1, St. Petersburg 199034, Russia; 2 Center of Rickettsioses and Arthropod-borne Diseases at the Infectious Diseases Department, Hospital San Pedro–Center for Biomedical Research of La Rioja, Logroño, Spain

**Keywords:** Harvest bug, Trombiculiasis, *Neotrombicula autumnalis*, *Neotrombicula inopinata*, Spain

## Abstract

**Background:**

For over a decade, the presence of trombiculid mites in some mountain areas of La Rioja (Northern Spain) and their association with seasonal human dermatitis have been recognized. This work aimed to establish the species identity of the agent causing trombiculiasis in the study area.

**Methods:**

Trombiculid larvae (chigger mites) were collected from vegetation in the Sierra Cebollera Natural Park and in Sierra La Hez during an outbreak of human trombiculiasis in 2010. Three specimens collected from a bird were also examined. Identification was made using morphological and morphometric traits based on the most recent taxonomic sources. A comparison of those mites with specimens of the same species collected throughout Europe was performed by means of cluster analysis with multiscale bootstrap resampling and calculation of approximately unbiased p-values.

**Results:**

All collected mites were identified as *Neotrombicula inopinata* (Oudemans, 1909). Therefore, this species is the most likely causative agent of trombiculiasis in Spain, not *Neotrombicula autumnalis* (Shaw, 1790), as it was generally assumed. No chigger was identified as *N. autumnalis* in the study area. *Neotrombicula inopinata* clearly differs from *N. autumnalis* in the presence of eight or more setae in the 1st and 2nd rows of dorsal idiosomal setae vs. six setae in *N. autumnalis*. Comparison of *N. inopinata* samples from different locations shows significant geographic variability in morphometric traits. Samples from Western and Eastern Europe and the Caucasus formed three separate clusters.

**Conclusion:**

Since the taxonomical basis of many studies concerning *N. autumnalis* as a causative agent of trombiculiasis is insufficient, it is highly possible that *N. inopinata* may be hiding behind the common name of “harvest bug” in Europe, together with *N. autumnalis*.

## Background

Nowadays, four chigger mite species are proven to attack humans in Europe causing trombiculiasis – *Neotrombicula autumnalis* (Shaw, 1790), *Kepkatrombicula desaleri* (Methlagl, 1928), *Blankaartia acuscutellaris* (Walch, 1922), and *Trombicula toldti* Winkler, 1953 [1]. The last one is a mite of unclear taxonomic position, which is still known solely from its type locality in Austria [2]. *Blankaartia acuscutellaris* is associated with reedy margins of water bodies (rivers, lakes, swamps, etc.). This widespread species principally infests birds inhabiting such biotopes, and humans are occasional hosts [1]. *Kepkatrombicula desaleri* parasitizes ungulates and has only been recorded on humans by the author who originally described it [3]. Unlike most trombiculids, this species is characterized by the presence of a sucker disk in the apical part of the hypostome that allows it to feed successfully on large hosts [4].

*Neotrombicula autumnalis* has been reported as the most frequent causative agent of human trombiculiasis in Europe [5-10], and its role as “harvest bug” has been attributed without sufficient taxonomic criteria [11,12]. To date, the harvest bug’s taxonomy remains unclear. Thus, Richards distinguished several forms of this species when designating the neotype of *N. autumnalis*[12]. Later, Kepka raised them to the subspecies level and equated two of them with the previously described taxa, *Neotrombicula inopinata* (Oudemans, 1909) and *Neotrombicula vernalis* (Willmann, 1942) [13]. Nowadays, both have been assigned the species rank [14-17]. Therefore, it is highly possible that the common name of “harvest bug” comprises more than one trombiculid species in Europe.

*Neotrombicula autumnalis* has been found in different locations in northern Spain [18-20]. Thus, cases of human and canine trombiculiasis have been recorded annually in La Rioja (North of Spain) from late summer to mid-autumn [21,22]. Dermatitis associated with chigger bites has repeatedly been a cause of public health concern in La Rioja, and in 2008 a study of trombiculiasis was financially supported by the Regional Government. One of the aims of this project was to correctly identify the chiggers present in this Region, since all cases had been ascribed to *N. autumnalis* despite no proper taxonomic identification ever having been carried out. Trombiculid larvae were collected in areas where the presence of mites and seasonal dermatitis cases in humans had been previously confirmed [21]. The collection of questing chiggers from vegetation allowed us to suggest the possible causative agent of trombiculiasis in the study area.

## Methods

### Collections

Chigger mites were collected during the scheduled samplings of a three-year study (2008–2010) by members of the Infectious Diseases Department, Center for Biomedical Research of La Rioja. A proportion of the chigger specimens collected on randomly-selected sample days were included in this study. According to the seasonality of human trombiculiasis in La Rioja, field collections were carried out from September to November 2010. Unfed trombiculid larvae ready to seek hosts were collected as follows:

1. The black-plate method, which is commonly used when collecting free trombiculid larvae from the ground, was combined with the flag technique, used to collect ticks on vegetation. Thus, a 20×40 cm black plastic plate with two strings at opposite corners was dragged over the ground. Chiggers were transferred into tubes containing distilled water with the aid of a brush.

2. By direct capture: chiggers on the upper surface of the leaves or on small dry branches were directly collected into tubes.

Additionally, three chiggers were removed with a swab from an *Erithacus rubecula* (L.) specimen captured during a bird ringing campaign carried out in the same area by Aranzadi Sciences Society and Abies Environment Resources Inc.

### Chigger collection sites

**Figure 1:**
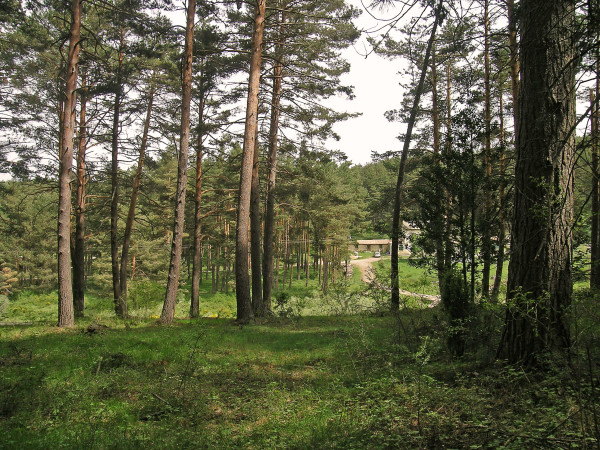
Collection site in La Pineda.

**Figure 2:**
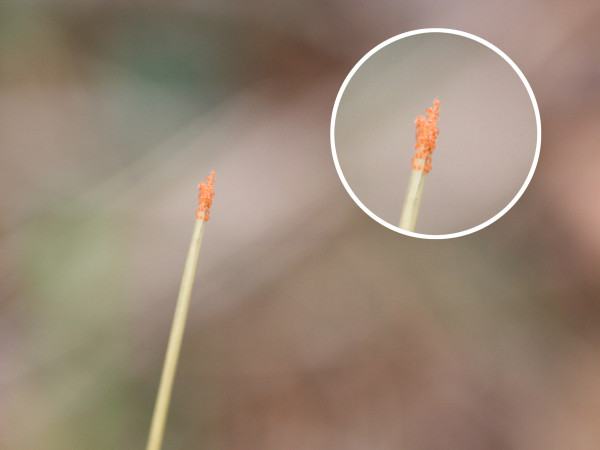
Cluster of unfed chigger mites at the end of a grass stalk, La Pineda.

**Figure 3:**
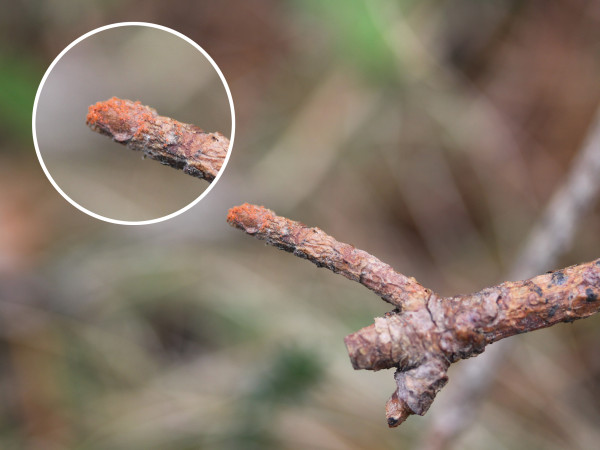
**Cluster of unfed chigger mites on a dried branch of *Pinus sylvestris*, La Pineda.**

Three sampling areas in La Rioja (North-West of the Central Iberian System, North of Spain) were chosen according to the proven existence of human and canine trombiculiasis:

1. La Pineda, 42º 06′ 00″ N, 2º 33′ 00″ W. It is located in Sierra Cebollera Natural Park, at 1,270–1,330 m above sea level (a.s.l.) (Figure 1). This location belongs to the municipal district of Lumbreras, 2.5 km away from the nearest village, San Andrés. Collections were made on 14th and 19th September, 4th and 11th November 2010 in a grove of *Pinus sylvestris* L. with undergrowth of *Erica vagans* L., *Ilex aquifolium* L., *Prunus spinosa* L., *Juniperus communis* L., and *Rubus fruticosus* L. Most chiggers were found on *Brachypodium* sp. at 15–40 cm above the ground (Figure 2) or on defoliated and dried branches of *P. spinosa* and *P. sylvestris* lying on the ground, at a height of 25–50 cm (Figure 3). Collection of chiggers from a bird was carried out in this location on 19th September, 2010.

2. Valdecalvo, 42º 07′ 00″ N, 2º 31′ 30″ W. It is located 4 km away from San Andrés and *c.* 2–3 km away from the previous location, at 1,400 m a.s.l. The vegetation has the same characteristics as described above for La Pineda. Sampling was performed on 4th November 2010.

3. Ocón, 42º 17′ 15″ N, 02º 14′ 30″ W. This place is located in the Sierra de La Hez (Leza-Jubera Basin). The collection site is located 2 km away from Ocón and *c.* 33 km away from previous locations, at 1,050 m a.s.l. Chiggers were collected on 7th October, 2010 in a *Quercus pyrenaica* Willd. grove with undergrowth of *Cytisus scoparius* (L.) Link, *Pteridium aquilinum* (L.) Kuhn, *Brachypodium* sp., and *Rubus fruticosus*.

**Figure 4:**
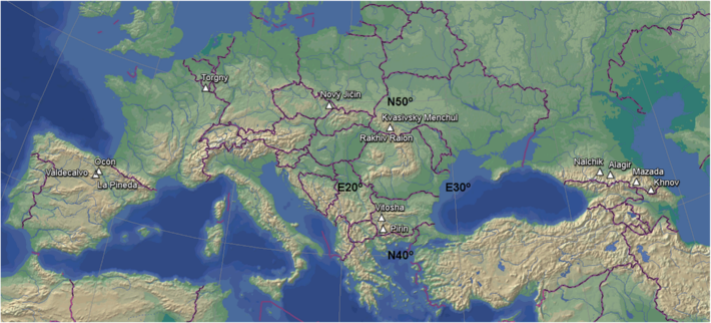
**Collection sites for *Neotrombicula inopinata*.**

For morphological comparison, *N. inopinata* samples representing widely separated parts of Europe and preserved as microscopic slides in different depositories were used (Figure 4):

1. Two specimens (Institut Royal des Sciences Naturelles de Belgique, Bruxelles) from *Glis glis* (L.), Belgium, Torgny, 49° 30′ N, 5° 28″ E, 1955, coll. A Fain.

2. Ten specimens [Zoological Institute RAS (ZIN), St. Petersburg, Russia] from *Prunella modularis* (L.), *Sylvia atricapilla* (L.), and *E. rubecula,* Czech Republic, Nový Jičin, 49° 34’ N, 17° 59′ E, 31.07–15.08.2005, coll. I Literák [23].

3. Six specimens [Zoological Museum of Moscow University (ZMMU), Moscow, Russia] from *Myodes glareolus* (Schreber), Ukraine, Transcarpatia, Rakhiv Raion, Kvasivsky Menchul Mt., 48° 10′ N, 24° 20′ E, 8.09.1959, coll. SO Vysotzkaja, identified as *Neotrombicula germanica* (Willmann, 1952) [24].

4. Ten specimens (ZMMU) from *M. glareolus*, Ukraine, Transcarpatia, Rakhiv Raion, coordinates unknown, 5.09.1959, coll. SO Vysotzkaja, identified as *N. germanica*[24].

5. Nine specimens (ZMMU) from *M. glareolus*, Bulgaria, Pirin Mts, 41° 46′ N, 23° 24′ E, 21–22.10.1960, coll. L Hristov, identified as *N. germanica*[25].

6. One specimen (ZMMU) from *M. glareolus*, Bulgaria, Vitosha Mts, 42° 34′ N, 23° 17′ E, 7.11.1960, coll. G Markov, identified as *N. germanica*[25].

7. Ten specimens (ZIN) from *Sorex raddei* Satunin and *Apodemus (Sylvaemus)* sp., Russia, Kabardino-Balkaria, Nalchik, 43° 26′ 20″ N, 43° 35′ 47″ E, 24.06.1996, coll. AA Stekolnikov.

8. Seven specimens (ZIN) from *Microtus* sp., Russia, North Ossetia-Alania, Alagir, 43° 02′ N, 44° 13′ E, 18.04.1976, coll. SN Rybin.

9. Nine specimens (ZIN) from *Cricetulus migratorius* (Pallas), *Apodemus (Sylvaemus)* sp., and *Chionomys gud* (Satunin), Russia, Dagestan, Mazada, 42° 11′ 40″ N, 46° 22′ 26″ E, 11–12.07.1988, coll. AB Shatrov.

10. One specimen (ZIN) from *Apodemus (Sylvaemus)* sp., Russia, Dagestan, 6 km E from Khnov, 41° 21′ 29″ N, 47° 31′ 36″ E, 22.06.1988, coll. AB Shatrov.

### Preparation and examination

Specimens from La Rioja selected for this work were fixed in 70% ethanol; 87 specimens were then mounted on microscopic slides in Faure-Berlese medium under uniform conditions. Clarified preparations were examined under a compound microscope MBI-3 (LOMO plc, St. Petersburg, Russia) with phase contrast optics. Measurements were made with an ocular micrometer and converted to micrometers. Digital photographs of mites were acquired with a Leica DM5000B microscope equipped with a DFC320 digital camera (Leica Microsystems Wetzlar GmbH), using differential interference contrast microscopy. All examined specimens are archived at ZIN (Nos 8250–8336).

### Statistical evaluation

The bootstrapping cluster analysis was used to estimate relative morphometric closeness between *N. inopinata* collected in La Rioja and samples from other regions. The analysis was based on the following 17 characters: AW (distance between anterolateral scutal setae), PW (distance between posterolateral scutal setae), SB (distance between sensillary bases), ASB (distance from the level of sensillary bases to extreme anterior margin of scutum), PSB (distance from the level of sensillary bases to extreme posterior margin of scutum), P-PL (distance from the level of posterolateral scutal setae to extreme posterior margin of scutum), AP (distance from anterolateral to posterolateral scutal setae on one side), AM (length of anteromedian seta of scutum), AL (length of anterolateral setae of scutum), PL (length of posterolateral setae of scutum), H (length of humeral idiosomal setae), pa (length of leg I, including coxa), pm (length of leg II, including coxa), pp (length of leg III, including coxa), DS (number of dorsal idiosomal setae), VS (number of ventral idiosomal setae), and dmt (distance from the base of leg III tarsus to the base of mastitarsala). Leg lengths were divided by 10 in order to equalize the magnitude of their variation with other variables [26].

Specimens with data missing for any variable were excluded from the analysis. The single specimen from Vitosha Mts was found to be an outlier and therefore was excluded. Lastly, the number of OTUs (Operational Taxonomic Units) was 12, and their sizes were as follows: La Pineda 10, Valdecalvo 5, Ocón 9, Torgny 2, Nový Jičin 10, Kvasivsky Menchul 6, Rakhiv Raion 10, Pirin 8, Nalchik 10, Alagir 7, Mazada 5, and Khnov 1. Mean values were calculated for each of them. Then the UPGMA clustering algorithm (Unweighted Pair Group Method with Arithmetic Mean) applied to the matrix of Euclidean distances between 12 OTUs by the above 17 variables was used. The number of bootstrap replications in the analysis was 1000.

The computations were performed using pvclust module [27] in the “R” software environment ver. 2.14.1 [28]. The package provides AU (approximately unbiased) p-values [29,30] and BP (bootstrap probability) p-values. For a cluster with AU p-value > 0.95, the hypothesis that “the cluster does not exist” is rejected with significance level 0.05 and such clusters can be considered as strongly supported by data [31]. We can also conclude, from our experience [26], that AU p-values slightly lesser than the threshold may also characterize well-delineated clusters.

## Results and discussion

**Figure 5:**
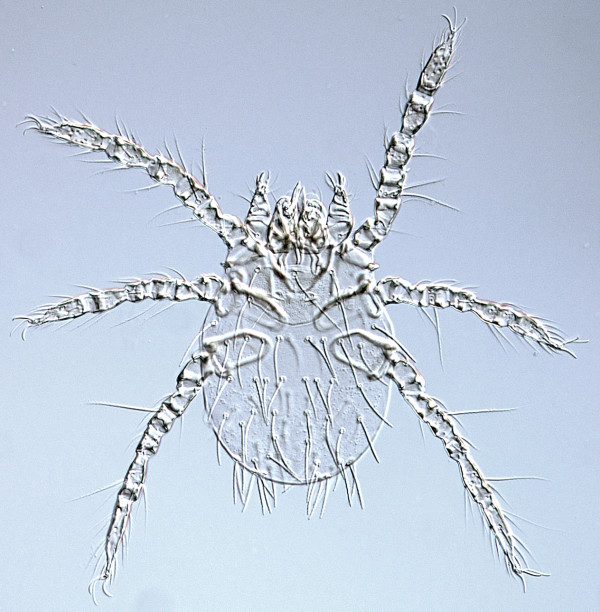
***Neotrombicula inopinata*, general view.**

**Figure 6:**
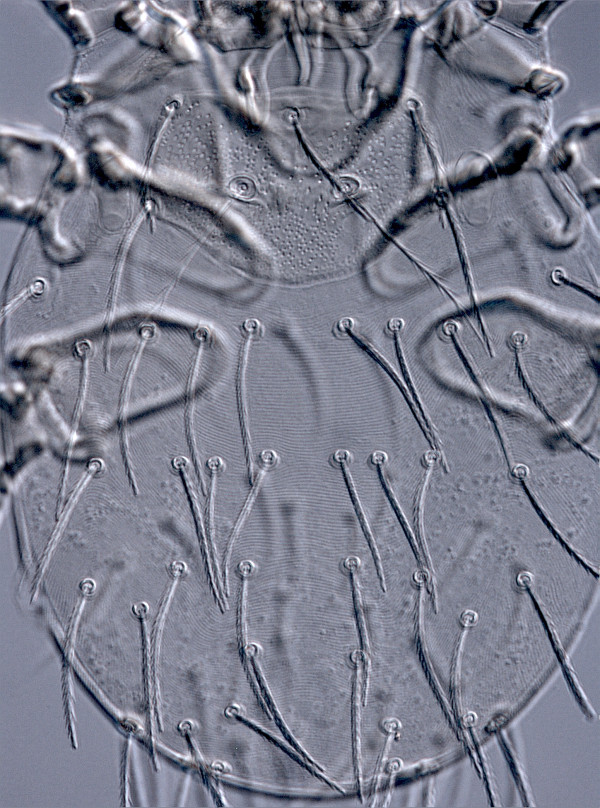
***Neotrombicula inopinata*, dorsal aspect of idiosoma.**

**Figure 7:**
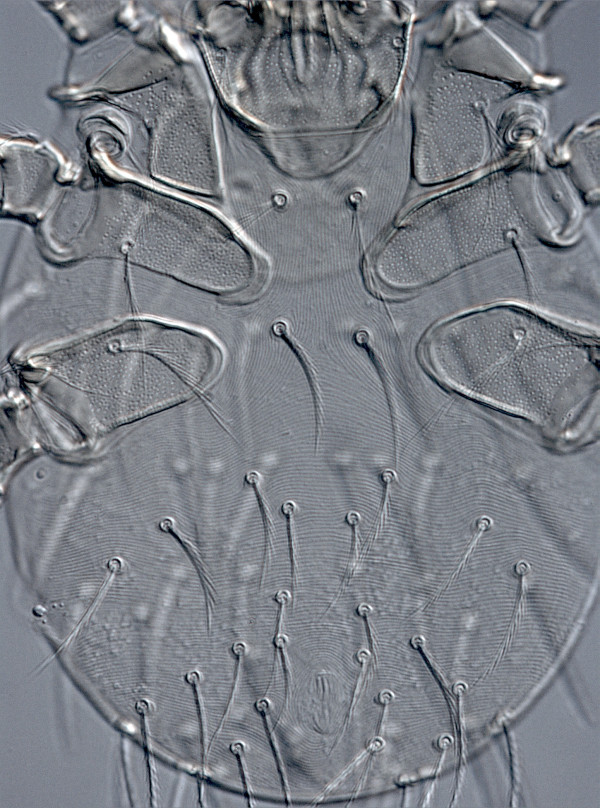
***Neotrombicula inopinata*, ventral aspect of idiosoma.**

All 87 examined specimens from La Rioja, including 84 collected from vegetation (54 in La Pineda, 15 in Valdecalvo, and 15 in Ocón) and 3 from the bird, were identified as *N. inopinata* (Oudemans, 1909) (Figures 5, 6 and 7). This species was described from Germany (Bremen) and later recorded also from England, France, Austria, Yugoslavia, Bulgaria, Ukraine, Russia (Northern Caucasus) [15], Romania [32], Hungary [33], Slovakia [34], Czech Republic [35], and Poland [36]. Here, we describe for the first time *N. inopinata* from Spain. The identification was achieved using appropriate descriptions and figures [15]. This species is characterized by the nude galeal seta (situated on the hypostome lobe between the cheliceral blade and the palp on each side), branched setae on palpal femur and genu, nude dorsal and lateral setae on palpal tibia, branched ventral palpal tibial seta, three genualae and one microgenuala I (specialized nude setae on leg genu), presence of a long nude seta on the leg tarsus III (mastitarsala), two humeral setae (most anterior marginal dorsal idiosomal setae) and 36–45 other dorsal idiosomal setae arranged as (6–11)-(8–13)-(6–10)-(2–10)-(2–9)-(0–6), with typical variant 8-8-8(6)-4-6-2 (Figure 6). The characteristic traits of the genus *Neotrombicula* Hirst, 1925 are available from several sources [14,17,37].

**Figure 8:**
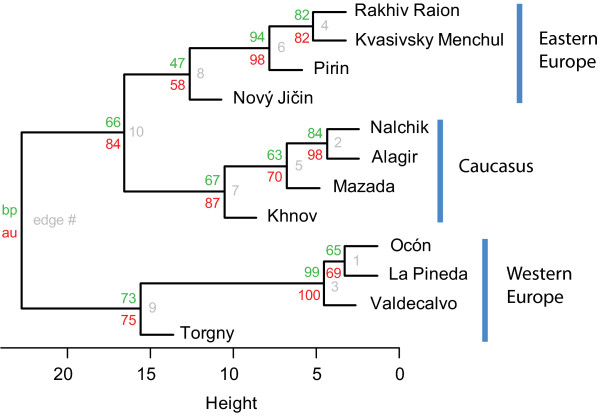
**Results of cluster analysis.** Au (numbers below), approximately unbiased p-values; bp (numbers above), bootstrap probability p-values; edge # (numbers right), sequence of cluster construction.

The comparison of the sample from La Rioja with specimens from the other regions (Table 1) showed that the former was distinguished first of all by shorter dmt and, consequently, smaller m-t (ratio dmt/TaIII where TaIII is a length of leg III tarsus), by a lower number of ventral idiosomal (VS) (Figure 7) and therefore all idiosomal setae (NDV = DS + VS), and by shorter anteromedian seta of scutum (AM). That these differences represent intraspecific variation is evident from their comparison with the ranges of variation in other widely distributed *Neotrombicula* species [38-40]. Moreover, a concordance between morphometric and geographic distances among OTUs can be clearly seen from Figure 8, where three clusters with significant statistical support (AU p-values > 0.95) correspond to groups of geographically closest samples (three OTUs from Spain, two from Caucasus, and three from Ukraine plus Bulgaria). It is noteworthy that upper-level grouping of all 12 OTUs, although without the same support, follows the geographical (meridian) sequence of sample sites (Figure 4), creating three large clusters consisting of Western European, Eastern European, and Caucasian samples, respectively.

**Table 1:** **Standard measurements of *Neotrombicula inopinata***

	**La Rioja (n = 24)**		**Other sites (n = 65)**	
**Variable**	**Range**	**Mean**	**Range**	**Mean**
AW	68–78	73	71–82	77
PW	82–93	88	86–104	95
SB	26–33	31	31–41	35
ASB	29–32	31	29–38	33
PSB	27–32	30	24–34	31
SD	56–63	60	54–69	64
P-PL	23–30	26	21–32	29
AP	27–33	30	25–36	31
AM	41–56	45	43–65	57
AL	38–46	43	40–49	45
PL	52–61	56	54–74	62
S	68–81	75	63–101	85
H	54–63	57	54–77	63
D_min_	38–49	42	36–50	44
D_max_	50–58	54	50–67	58
V_min_	29–36	33	26–38	34
V_max_	41–48	45	43–65	52
pa	310–337	324	274–331	300
pm	265–295	282	248–299	270
pp	306–335	320	277–342	310
Ip	887–963	925	808–967	880
DS	36–44	38	36–45	40
VS	22–30	26	23–47	36
NDV	60–72	64	66–90	77
TaIII	77–86	82	67–85	76
dmt	9–15	11	11–21	17
m-t	0.109–0.177	0.139	0.138–0.288	0.220

The fauna of trombiculids in Spain, based on collection of chiggers from small mammals, reptiles and birds included 18 species, according to Kepka [18,41], Varma [42], Pereira-Lorenzo [19], and Imaz *et al.*[20]. In particular, 10 trombiculid species were recorded on *Apodemus sylvaticus* (L.) from Navarra, a region bordering La Rioja [20]. The description of *N. inopinata* collected from vegetation in La Rioja has raised to 19 the number of trombiculid species in Spain:

*Blankaartia acuscutellaris* (Walch, 1922),

*Hirsutiella billabeta* Imaz, Galicia, Moraza et Stekolnikov, 2006,

*H. zachvatkini* (Schluger, 1948),

*Xinjiangsha tarda* (Schluger, 1957),

*Neotrombicula autumnalis* (Shaw, 1790),

*N. inopinata* (Oudemans, 1909),

*N. hispanica* Kepka, 1960,

*N. japonica* (Tanaka, Kaiwa, Teramura et Kagaya, 1930),

*N. jordana* Imaz, Galicia, Moraza et Stekolnikov, 2006,

*N. vulgaris* (Schluger, 1955),

*Ericotrombidium hasei* (Feider 1958),

*Leptotrombidium europaeum* (Daniel et Brelich, 1959),

*L. silvaticum* Hushcha et Schluger, 1967,

*Brunehaldia bulgarica* Vercammen-Grandjean et Kolebinova, 1966,

*Cheladonta ikaoensis* (Sasa, Sawada, Kano, Hayashi et Kumada, 1951),

*C. pannonica* (Kepka 1956),

*Helenicula olsufjevi* (Schluger, 1955),

*Schoutedenichia dipodilli* Vercammen-Grandjean, 1958,

*S. krampitzi* (Willmann, 1955).

To collect chiggers from vegetation is of primary importance in order to find the agent causing human trombiculiasis. Chigger mites located on grass, shrubs or low tree branches are unfed larvae ready to attack their hosts. Chigger infestation in humans is very probable due to direct contact with these substrates during excursions, hiking or working. All human cases of trombiculiasis recorded in La Rioja have been diagnosed in people that had been in contact with the vegetation from Sierra Cebollera Natural Park during the season when the peak of abundance of *N. autumnalis* and related species is usually recorded in Europe [6,14,43]. Affected people do not detect the presence of trombiculid mites. In fact, chigger bites are noticed later on, due to the associated intense itching. By then, the mites are detached and examination of the patients skin does not reveal any “harvest bug”. The association between chigger mites and human trombiculiasis in the study area is only based on epidemiological evidence. In accordance with this, collections of chiggers from vegetation have been made previously in Spain [21,44]. All collected chiggers have been identified as *N. autumnalis* but with insufficient taxonomic evidence. Since *N. autumnalis* is closely related to *N. inopinata*, the characteristics that distinguish these species should be examined in each case in order to confirm the identification. *Neotrombicula inopinata* clearly differs from *N. autumnalis* by the presence of eight or more setae in 1st and 2nd rows of dorsal idiosomal setae (Figures 5 and 6), rarely six or seven setae in one of these rows, while *N. autumnalis* always has six setae in both rows. Accordingly, the number of dorsal idiosomal setae is 36–45 in *N. inopinata* and 26–33 in *N. autumnalis*. Futhermore, *N. inopinata* is slightly larger than *N. autumnalis* (larger scutum, longer setae, and longer legs), but intervals for individual morphometric traits overlapped when our entire sample was compared to specimens of *N. autumnalis* collected throughout its geographic range [38].

It should be noted that taxonomical basis of many studies concerning trombiculiasis is inadequate or nonexistent. A notable case was the record of *N. autumnalis* on a little bittern from Turkey [45], where no taxonomic evidence was provided. In fact, the existence of this species in Turkey is not confirmed [17]. Occasionally, when authors try to support their identifications by morphological data, these are surprisingly inappropriate. For example, Guarneri *et al.*[46] provided some descriptions in order to justify their record of *N. autumnalis* in Italy. Their method included traits common to larvae of several mite families, such as three pairs of legs with three claws, paired eyes, hooked chelicerae, segmented palps, and some of the typical traits shared by many chigger mite genera such as pentagonal scutum with five setae and two sensilla, nude galeal seta, and tricuspid palpal claw. Kampen [9] treated *N. inopinata* and *N. vernalis* as “subspecies or ecotypes” of *N. autumnalis* following old papers of Kepka [13,47], ignoring all subsequent development of chigger mite taxonomy. Schöler *et al.*[10] stated that the only species involved in their investigation is *N. autumnalis*. Nevertheless, only outdated references [13] and no information about the identification process are included. In the light of these facts, most published data concerning *N. autumnalis* as the causal agent of trombiculiasis should be considered as doubtful. A confirmation of the role of this species in human trombiculiasis, based on new collections and appropriate examination, is required.

In Spain, human trombiculiasis has been only described in the study area [21], and no cases have been reported from the remainder of the country. According to our data, *N. inopinata* is the only chigger species on vegetation during the seasonal outbreak of trombiculiasis. Therefore, it is the only candidate for the causative agent of trombiculiasis in this territory. However, we cannot conclude that *N. inopinata* is the only mite of medical and veterinary importance in northern Spain. To date, no other trombiculid species has been found in La Rioja, although additional chigger collections from birds and small mammals may increase the trombiculid fauna of the region, especially with the species previously recorded in Navarra [20]. Thus, it is highly probable that *N. autumnalis* parasitizes small mammals in the study area, although its role as causative agent of trombiculiasis is unclear. The different height of locations where unfed chiggers wait for their hosts (e.g. vegetation, low tree branches or forest floor), could result in the different host spectra of diverse trombiculid species [39,48]. According to our results, since *N. autumnalis* has not been collected from vegetation, it would be unlikely to attack humans or large animals, whereas *N. inopinata* would have a privileged position to infest humans. The identification of engorged trombiculid mites that have developed their stylostomes, directly removed from humans or domestic animals is essential to define the etiological agent of trombiculiasis.

## Conclusion

*Neotrombicula inopinata* is the most likely causative agent of trombiculiasis in northern Spain, rather than *N. autumnalis,* the species usually regarded as the main human-attacking chigger mite in Europe. Taking into consideration that *N. inopinata* is widely distributed in Europe, this species could be hidden behind the common name of “the European harvest bug” together with *N. autumnalis,* or even exceed the latter in medical and veterinary importance.

## Competing interests

The authors declare they have no competing interests.

## Authors’ contributions

Collected material, made photographs of the collection site and chigger clusters: PS, AMP, JAO. Prepared and identified material, made measurements and microphotographs, performed statistical evaluation, analyzed the data: AAS. Wrote the paper: AAS, PS, AMP, JAO. All authors read and approved the final version of the manuscript.
